# Consensus-based Detection of Aetiologic Copy Number Variants For Syndromic Orofacial Clefts Utilising Whole Exome Sequencing of Case Parent Trios

**DOI:** 10.21203/rs.3.rs-9225341/v1

**Published:** 2026-04-01

**Authors:** Samuel Kanor Quaynor, Gideon Okyere Mensah, Tamara Busch, Bruce Tsri, Solomon Obiri-Yeboah, Daniel Kwesi Sabbah, Pius Agbenorku, Peter Donkor, Azeez Butali, Lord Jephthah Joojo Gowans

**Affiliations:** Kwame Nkrumah University of Science and Technology (KNUST); Kwame Nkrumah University of Science and Technology (KNUST); University of Iowa; Kwame Nkrumah University of Science and Technology (KNUST); Kwame Nkrumah University of Science and Technology; Kwame Nkrumah University of Science and Technology; Kwame Nkrumah University of Science and Technology; Kwame Nkrumah University of Science and Technology; University of Iowa; Kwame Nkrumah University of Science and Technology (KNUST)

**Keywords:** Orofacial Clefts, Genetic syndromes, Copy Number Variants, Whole Exome Sequencing, Read Depth, Sub-Saharan Africans

## Abstract

**Background:**

Orofacial clefts (OFCs) are the most common craniofacial congenital anomalies, with complex aetiology involving both genetic and environmental factors. Most genetic studies on the condition have focused on the contribution of single nucleotide variants (SNVs) and small insertions and deletions (indels). However, the contribution of copy number variants (CNVs), especially in African populations, remains underexplored despite their known contribution to congenital anomalies. This study aimed to identify high-confidence CNVs contributing to the aetiology of syndromic OFCs in Ghanaian case parent trios using whole exome sequencing (WES) datasets.

**Methods:**

WES data from Ghanaian case parent trios were processed through a comprehensive five-phase pipeline. Following stringent quality control and preprocessing, CNVs were called using four independent tools, namely, cn.MOPS, CODEX, ExomeDepth, and GATK-gCNV. The called CNVs were merged through a consensus-based approach using BEDtools, requiring support from at least two tools to classify them as true CNVs. AnnotSV was used to annotate and classify CNVs, while VarElect was employed to prioritise CNVs based on clinical phenotypes. High-confidence CNVs were mapped to patient phenotypes and further interrogated for pathogenic potential using databases such as DECIPHER, ClinVar, Mouse Genome Informatics (MGI), and the Alliance of Genome Resources. Gene expression patterns utilized MGI, Zebrahub and CELLxGENE Discover. Finally, pathway enrichment and interaction analyses were performed using g:Profiler, the STRING database, and Cytoscape.

**Results:**

Several *de novo* and inherited CNVs were identified, including deletions and duplications involving key genes such as *SHH*, *WBP11*, and *ADAMTS2*, all of which are critically involved in craniofacial morphogenesis. In addition to known OFC-associated genes, the analysis identified novel CNV regions encompassing genes not previously linked to syndromic OFCs in humans, including *HYDIN*, *FLI1*, *ETS1*, *RSPH10B2*, and *CCZ1B*. These were prioritised based on their expression patterns in developmental models, suggesting potential functional relevance to OFC pathogenesis. Pathway enrichment analysis further identified significant biological processes associated with craniofacial, neurodevelopmental, and musculoskeletal development.

**Conclusion:**

This study highlights the value of CNV analysis in studies on the genetic aetiology of OFCs and supports broader inclusion of African genomic data to identify population-specific aetiologic variants, thereby enhancing understanding of pathophysiology and clinical care.

## INTRODUCTION

Orofacial clefts (OFCs) are among the most common congenital malformations, mostly characterised by an opening in the lip and/or palate that arises from incomplete fusion of facial prominences during embryonic development [1, 2]. These dysmorphologies may occur as non-syndromic, in which no other congenital anomalies are present, or as syndromic, in which additional clinical features or developmental anomalies accompany the cleft [3]. Globally, OFCs occur in approximately 1.7 per 1,000 live births [4]. In Africa, the estimated prevalence is roughly one in every 2,000 births [5], though these figures likely underestimate the true burden due to underreporting and limited surveillance [6]. In Ghana, reported prevalence ranges from 1.31 to 6.3 per 1,000 births, representing a considerable public health concern [7, 8]. The number of reported cases has increased in recent years, likely reflecting improved awareness and access to free reconstructive surgical services [9]. Beyond their visible impact, OFCs are associated with multiple complications, including feeding and nutritional challenges, speech and hearing impairments, and psychosocial and economic burdens on affected families [10].

The aetiology of OFCs is multifactorial, involving both genetic and environmental influences [11]. Syndromic forms, which account for roughly 30% of cases, often present with additional cardiovascular, neurodevelopmental, or musculoskeletal defects [12]. More than 500 syndromes associated with OFCs have been described, many of which arise from single-gene mutations or chromosomal abnormalities [13]. For example, pathogenic variants in *IRF6* cause Van der Woude syndrome (VWS), the most common Mendelian form of clefting, characterised by lower lip pits and dental anomalies [14]. Pierre Robin sequence (PRS), characterised by micrognathia, glossoptosis, and airway obstruction, is often associated with mutations in *SOX9*, *MAP2K6*, and *KCNJ2* [15, 16]. Similarly, mutations in collagen genes (*COL2A1*, *COL9A1*, *COL11A1*, and *COL11A2*) underlie Stickler syndrome, while chromosomal deletions such as the 22q11.2 deletion cause Velocardiofacial or DiGeorge syndrome [14].

While the contribution of single nucleotide variants (SNVs) and small insertions/deletions (indels) to OFCs has been widely studied, large-scale structural variations, particularly copy number variants (CNVs), remain underexplored [17], especially in African populations. A systematic review of genetic studies conducted in Africa between 2000 and 2020 identified 35 novel variants associated with OFCs, highlighting the immense genetic diversity of African populations and their usefulness in identifying etiological loci [5]. However, most African OFC studies have focused on smaller genetic variants, overlooking CNVs, which are known to disrupt coding regions, influence gene dosage, and alter gene expression levels [18]. This gap in CNV research limits our understanding of the broader genomic architecture underlying OFCs in African cohorts.

Whole Exome Sequencing (WES) has gained popularity due to its focus on exonic regions, which harbour approximately 85% of disease-causing mutations [19]. Compared to Whole Genome Sequencing (WGS), WES is less costly, easier to interpret, and has a higher diagnostic yield [20]. Given that many disease-associated CNVs span coding regions, WES provides an attractive option for CNV detection [21]. Numerous algorithms capable of detecting CNVs from WES data have emerged. Most of these tools infer copy number changes by estimating and analysing read depth fluctuations across exons [22].

Given the genetic heterogeneity of OFCs and the underrepresentation of African populations in global genomic research, it is crucial to investigate the role of CNVs in the aetiology of these disorders. To our knowledge, no study has employed WES to systematically detect and characterise CNVs in syndromic OFC patients from African populations. Addressing this gap will advance the understanding of structural genomic variation in OFCs and provide a foundation for genetic counselling and translational applications in clinical genetics in African populations. Therefore, this study aimed to adopt a consensus-based approach to detect, annotate, and prioritize high-confidence CNVs associated with syndromic OFCs using whole exome sequencing of Ghanaian case-parent trios, and to evaluate their potential relevance to craniofacial development. We hypothesized that utilizing a consensus-based approach would enable the assessment of the strengths of four CNV calling tools for calling CNVs from WES datasets, characterize unique and overlapping CNVs called by the tools, and functionally annotate the CNVs. This approach helps make the most of WES data in a setting where many genomic services, including those for calling structural variants such as CNVs, are lacking.

## MATERIALS AND METHODS

### Study cohort and ethical approval

The study included 26 case-parent trios, each comprising a proband with syndromic OFC and their unaffected parents, for a total of 78 individuals. Data on the clinically observed phenotypes of the probands were also obtained. Ethical approvals were obtained from Kwame Nkrumah University of Science and Technology (KNUST) Institutional Review Board, IRB (CHRPE/RC/018/13) and Komfo Anokye Teaching Hospital, KATH IRB (KATH-IRB/AP/032/20). Participants aged 18 or older provided written informed consent, whereas for those under 18, parents or legal guardians provided consent.

### Whole exome sequencing dataset

The WES dataset was obtained from the Human Genetics and Genomics (HuGENE) Laboratory at KNUST and is available through FaceBase under Accession Number 94-D420. A brief summary of how the data was generated is given here. Saliva and buccal swab samples were collected from participating families using the Oragene DNA Saliva Collection Kit (DNA Genotek, Ottawa, ON, Canada). Genomic DNA was extracted and purified following a protocol adapted from the Oragene saliva DNA extraction method, as previously described [23]. Extracted DNA underwent quality control assessments, including concentration measurement, purity evaluation, and XY genotyping to genetically confirm sample sex. DNA quantification was performed using the Qubit^™^ dsDNA Broad Range (BR) and High Sensitivity (HS) assays on the Qubit^™^ 4.0 Fluorometer (Thermo Fisher Scientific, Waltham, MA, USA) [23].

Whole exome sequencing (WES) library preparation and sequencing were carried out by Azenta Life Sciences (USA), targeting an average read depth of 100X. Prior to library preparation, DNA samples were re-quantified using the Qubit 4 Fluorometer. Exome capture libraries were prepared using the Twist Human Comprehensive Exome kit according to the manufacturer’s instructions. Genomic DNA was fragmented using a Covaris S220 ultrasonicator, followed by end repair, A-tailing, and adapter ligation. Libraries were PCR-amplified, quality-checked using the Agilent TapeStation, and hybridized with biotinylated capture probes. Targeted DNA fragments were enriched using streptavidin-coated magnetic beads, followed by post-capture amplification and indexing with Illumina-compatible primers [24].

Sequencing was performed on an Illumina HiSeq platform using a 2 × 150 bp paired-end configuration across multiple flow-cell lanes. Image analysis and base calling were conducted using HiSeq Control Software (v2.2.68), generating binary base call (BCL) files. These files were converted to FASTQ format and demultiplexed using Illumina bcl2fastq v2.19, allowing for one mismatch in index identification [24].

### Bioinformatics analysis of whole exome sequencing datasets

**Figure 1:**
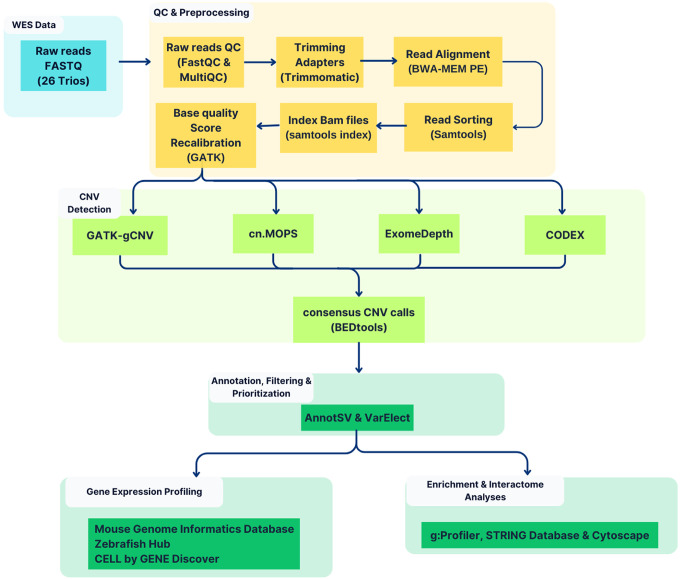
Overall scheme of consensus-based CNV detection employed in the study. After quality control and preprocessing, consensus results from GATK-gCNV, cn.MOPs, ExomeDepth, and CODEX were merged based on a 50% reciprocal overlap criterion. The Consensus CNVs were then annotated, filtered and prioritised for further downstream analyses.

The bioinformatics analysis in this study was structured into five main phases (Fig. 1). The first phase involved quality checks and preprocessing of raw sequencing data. The second phase involved detecting CNV using WES-based algorithms. In the third phase, the identified CNVs were annotated and prioritised. The fourth stage involved gene expression profiling of highly prioritised genes in the high-confidence CNV regions. Enrichment and interaction analyses were conducted at the final stage.

### Quality control and preprocessing

To ensure high-quality data for downstream analysis, the raw FASTQ files were assessed using FASTQC v0.12.1 (https://www.bioinformatics.babraham.ac.uk/projects/fastqc/, 17 December, 2025). After generating individual FASTQC reports, MultiQC v1.25 [25] was used to aggregate them into a single comprehensive summary. Trimmomatic v0.39 [26] was used to trim low-quality bases and technical sequences, such as adapters, to avoid inefficient and subpar downstream analysis. The trimmed reads were mapped to the human reference genome hg38 to determine genomic locations. The hg38 reference genome was downloaded from the UCSC Genome Browser’s analysis set files (https://hgdownload.soe.ucsc.edu/goldenPath/hg38/bigZips/analysisSet/, 17 December, 2025), which is a version optimised for NGS read alignment. BWA-MEM v0.7.18 [27], a widely used alignment algorithm known for its accuracy with high-quality paired-end reads, was used to align the reads to the human reference genome file hg38.fa. Samtools v1.21 [28] was used to convert the aligned data from SAM (Sequence Alignment/Map) to BAM (Binary Alignment/Map) format, a format compatible with CNV detection tools. Post-alignment processing, including duplicate marking, reference genome preparation, and base quality score recalibration, was performed to prepare the data for variant calling.

### CNV detection using four different tools

Prior to CNV detection, the Twist Comprehensive Exome Capture bed file (https://www.twistbioscience.com/resources/data-files/comprehensive-exome-bed-files, 17 December, 2025) defining the capture targets or genomic regions for the WES data was downloaded. Based on the characteristics of WES data, four CNV detection tools — GATK gCNV, ExomeDepth, CODEX, and cn.MOPs, were chosen to call CNVs from the data. These algorithms were selected based on their suitability for detecting germline CNVs, ability to call CNVs without the need for unrelated control samples, evidence of widespread usage, active maintenance and availability of detailed user documentation. The rationale for selecting four CNV detection tools was to increase the accuracy of CNV calls, using a consensus-based approach that relies on CNVs called by at least two of the tools.

The GATK gCNV workflow [29] is primarily divided into model creation and CNV calling. During model creation, the cohort mode was specified using the entire dataset to generate a cohort model and then call CNVs for the cohort samples. Intervals were generated with a padding of 250 bases. Read counts were measured, and intervals were annotated with GC content and mappability scores. Intervals with outlier read counts and potentially confounding annotations were excluded. Baseline Ploidy models were then generated. Finally, a model per chromosome was produced using GermlineVariantCall. Chromosomal calls were finally merged using PostprocessGermlineCNVCalls.

For CNV calling using ExomeDepth, the vignette for ExomeDepth [30] was adapted with slight modifications for calling CNVs from our WES dataset. The initial step involved the generation of count data from the BAM files with subsequent annotations and filtering steps to exclude low-mappability regions. For each test sample, an optimised reference set made of the same gender as the test sample was constructed to improve CNV detection accuracy. The beta-binomial model was then applied across the dataset, and CNVs were called using a hidden Markov model.

To call CNVs with CODEX [31], the depth of coverage and read length were computed across all samples. A multi-sample normalisation model was used to establish a null model representing expected coverage in the absence of CNVs. Following normalisation, a Poisson-likelihood-based segmentation algorithm was applied to group contiguous regions with homogeneous copy numbers by comparing observed coverage with the normalised control coverage for each sample. The segmented regions were assessed to identify potential CNVs.

In CNV calling using cn.MOPs [32], read counts were first extracted from the BAM files and normalised by adjusting for each sample’s total number of mappable reads. The read count variation across samples was modelled to identify CNVs. Following segmentation, integer copy numbers were estimated, yielding discrete copy number calls for each identified segment.

### Generation of consensus CNV calls

To achieve high-confidence CNV calls, a consensus approach was implemented by integrating the outputs from multiple CNV calling tools. First, the CNV call files from each tool were standardised to BED format, facilitating genome arithmetic operations. For each sample, “BEDtools intersect” [33] was used to identify overlapping CNV regions across tools, with intersections defined by at least 50% reciprocal overlap at matching genomic coordinates. The 50% threshold was chosen based on thresholds from previous comparative research [34–37]. This intersection process was performed across all tool-generated CNV calls for each sample. Following the intersection, the intersected CNV calls were concatenated across tools into a unified file for each sample, streamlining further analysis. To finalise consensus CNV calls, “BEDtools merge” was applied to combine overlapping regions with the same CNV type. For each merged region, the number of tools supporting the call was counted, ensuring that only regions with support from at least 2 tools were retained as consensus CNVs.

### Annotation of CNVs with AnnotSV

To evaluate the pathogenic potential of the high-confidence CNV regions, functional annotation was carried out using AnnotSV [38]. The annotations included key genomic characteristics such as gene names, predicted pathogenicity, allele frequencies, and Exomiser scores. For each proband, Human Phenotype Ontology (HPO) terms representing the observed clinical phenotypes were provided as input, along with a BED file detailing the genomic coordinates and CNV type for each region. The output from AnnotSV included comprehensive annotations, with particular emphasis on the American College of Medical Genetics and Genomics (ACMG) classifications and Exomiser scores. These metrics were used to assess the likelihood that each CNV region was contributing to the observed phenotype.

### Prioritisation of genes within CNV regions with VarElect

To assess the relevance of genes within high-confidence CNV regions to patient phenotypes, VarElect [39] was used to prioritise genes with potential disease associations. Genes annotated by AnnotSV with an Exomiser score of at least 0.5 were selected for further analysis in VarElect. Each gene was evaluated for its association with the documented clinical phenotypes of the patients, utilising the comprehensive disease databases of VarElect.

### Gene expression profiling in mouse, zebrafish and human

To explore the developmental relevance of genes disrupted by high-confidence CNV regions, spatiotemporal gene expression analysis was performed using publicly available datasets from the Mouse Genome Informatics (MGI) Gene Expression Database (GXD), https://www.informatics.jax.org/expression.shtml [40].

Genes prioritised from CNV regions identified through WES analysis were queried using the Batch Search functionality within the GXD platform. This approach enabled systematic interrogation of multiple candidate genes simultaneously. Following query execution, gene-specific expression annotations were accessed through the Tissue × Gene Matrix view, which summarises expression evidence across tissues and developmental stages.

Analyses were restricted to Theiler stages T14–T28 corresponding to key developmental milestones in mouse embryos. Filters were applied to focus on the anatomical system *Conceptus*, with particular emphasis on craniofacial structures implicated in syndromic OFCs, including the branchial arches, maxillary processes, palatal shelf, secondary palate, mesenchyme derived from neural crest and nasal processes [41].

This integrative expression profiling approach was used to assess whether genes affected by CNVs exhibit biologically plausible expression patterns during key stages of craniofacial development, thereby supporting their potential contribution to the aetiology of syndromic OFCs. To further examine gene expression at the transcript level, expression intensity was assessed using the *Heat Map* visualisation available within the GXD interface. This view presents normalised gene expression values reported as transcripts per million (TPM), generated using the Morpheus visualisation platform developed by the Broad Institute (https://software.broadinstitute.org/morpheus/).

To decipher the expression patterns of genes in the implicated CNV regions at the cellular level, single cell transcriptomics data from zebrafish (10–72 hpf) were obtained from the Zebrahub atlas (https://zebrahub.sf.czbiohub.org/). We generated high-resolution UMAP visualizations and cell-type annotations, which enabled the identification of specific cell subpopulations that express the genes. This uncovers cell-type-specific gene expression patterns that may be masked by bulk gene expression data from the mouse. Gene expression patterns were ascertained in many structures of craniofacial importance, including periderm, mesenchyme, endoderm, neural crest, and mesodermal cells.

In order to ascertain if the observed expression patterns of the genes in mouse and zebrafish models recapitulate that of human post-natal craniofacial tissues, human single cell transcriptomics dataset was retrieved from the CELLxGENE Discover platform (https://cellxgene.cziscience.com). Gene expression patterns were obtained for critical structures relevant to craniofacial development, including epithelial, neural crest, endodermal, and mesodermal cells. Confirming gene expression in analogous human cell types gives credence to the biological relevance of the implicated genes to OFC etiology, potentially culminating in the translational significance of the implicated genes.

### Pathway enrichment analysis, protein-protein interaction network and hub genes identification

To gain insights into the biological significance of the identified CNV regions and their associated genes, we performed pathway enrichment analysis using g:Profiler [42]. The prioritised genes in CNV regions were uploaded to g:Profiler for overrepresentation analysis. Default settings were employed, including the use of the g:SCS (Set Counts and Sizes) algorithm to account for multiple testing corrections and ensure stringent statistical significance. The results were filtered to include only significantly enriched terms (adjusted p-value < 0.05). The resulting enriched terms were imported into Cytoscape v.3.10.4 [43] for visualisation. We applied the EnrichmentMap plugin v3.5 [44] to group related terms into functional clusters.

For the protein-protein interaction (PPI) analysis, we queried the STRING database [45] using the “multiple proteins” option with the full list of candidate genes to generate an interaction network. The network was then exported into Cytoscape for downstream processing. To pinpoint key central proteins, we employed the CytoHubba plugin v0.1 [46] to rank nodes based on the Maximal Clique Centrality (MCC) score.

## RESULTS

### Phenotypic characteristics of the study cohort

**Figure 2:**
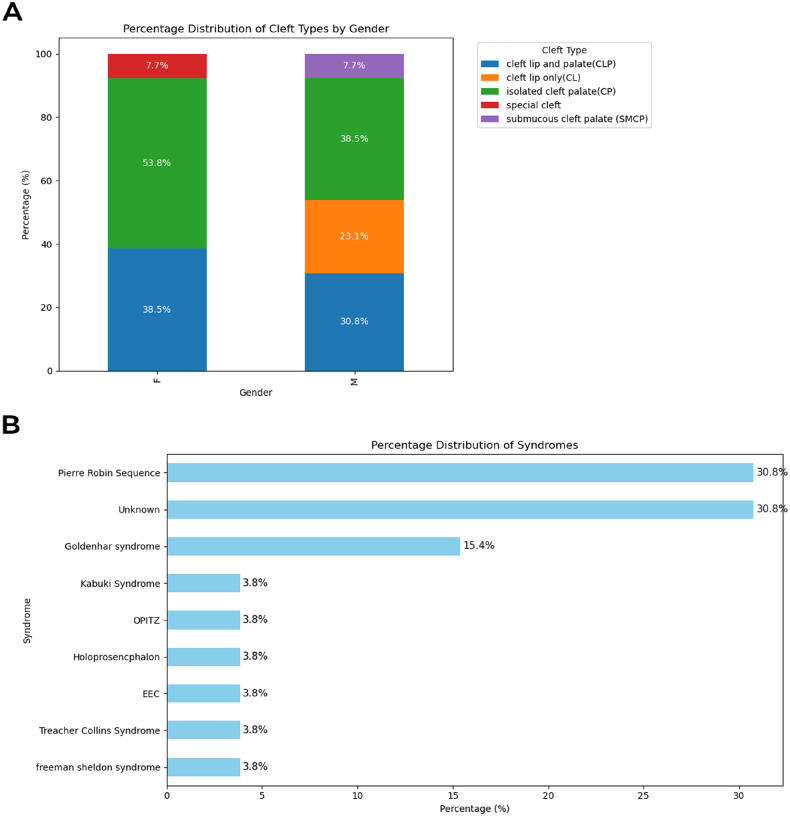
Orofacial cleft subphenotypes and suspected syndromes. **(a)** Distribution of clinically observed cleft subphenotypes across genders. F represents the female gender; M represents the male gender. **(b)**Distribution of clinically observed syndromes among affected probands. EEC represents ectrodactyly ectodermal dysplasia.

This study analysed 26 case-parent trios, comprising a total of 78 individuals. All probands were classified as syndromic OFCs, with equal sex distribution of affected males (n = 13) and females (n = 13). A range of OFC subphenotypes was observed in the cohort. As shown in Fig. 2a, cleft palate was the predominant OFC subphenotype among females (53.8%) as well as in males (46.2%). Only one female (7.7%) exhibited a facial cleft characterised as Tessier number 30, accompanied by a bifid tongue, low-set ears, and a soft-tissue cleft extending from the mandible to the jugular notch of the sternum.

The suspected genetic syndromes associated with the OFCs in the cohort, based on clinical evaluation, were diverse (Fig. 2b). Clinical classifications at the time of subject recruitment relied only on observable physical traits, not molecular data, due to the lack of genetic testing services in the study population. While some probands were assigned specific syndromes based on clinical evidence, others were simply described by their symptoms and classified as “unknown,” as the clinical evidence was insufficient to confidently assign a particular syndrome. Pierre Robin Sequence (PRS) represented the most frequently observed syndrome, accounting for 30.8% of probands. A detailed summary of phenotypic features for all probands is provided in Supplementary Table S1.

### Consensus-based CNV detection from the whole exome sequencing datasets

**Figure 3:**
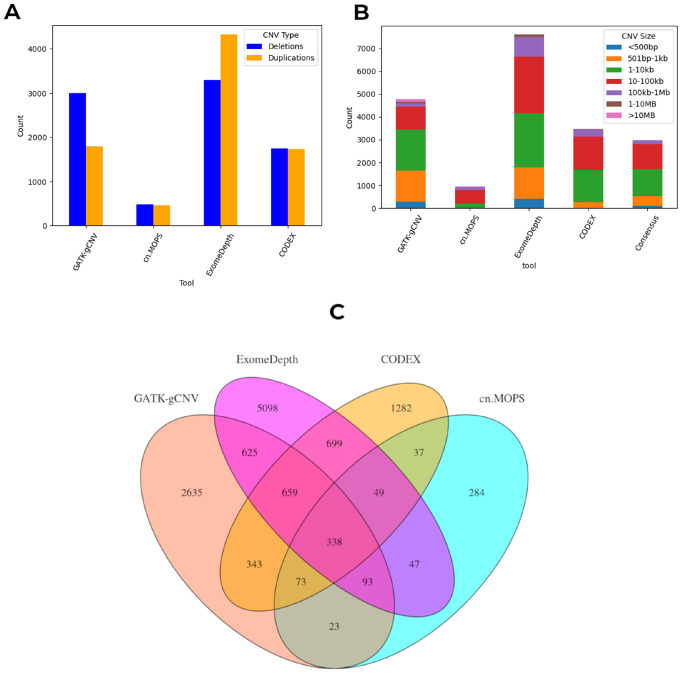
Diversity and sizes of copy number variants detected by various tools. **(a)** Counts of CNVs across tools for both deletions and duplications. **(b)** CNV size distribution between the four CNV calling tools. **(c)** Overlap in CNVs between the four CNV calling tools. A 50% overlap criterion was used for the intersection of CNVs across the tools.

Four WES-based CNV detection tools, namely GATK gCNV, ExomeDepth, CODEX, and cn.MOPs, were employed to identify CNVs in our cohort. Notably, all tools except ExomeDepth, detected a greater number of deletions than duplications (Fig. 3a). To evaluate potential tool-specific bias in CNV type, a paired Wilcoxon signed-rank test was performed, with results summarised in Supplementary Figures S1 and S2. The total number and size distribution of CNVs varied across tools (Fig. 3b). ExomeDepth identified the highest number of CNVs (7,608), while cn.MOPs detected the fewest (944). All tools successfully detected CNVs across a broad range of sizes, though cn.MOPs was unable to identify variants smaller than 1 kb. Across tools, CNVs within the 1–100 kb range were most abundant. To obtain high-confidence CNV calls, we performed an intersection analysis to identify CNVs detected by at least two tools. After the intersection analysis, 2,980 high-confidence CNVs were retained. cn.MOPs exhibited the highest overlap rate (69.9%), while ExomeDepth had the lowest (33.0%). The consensus CNVs derived from this approach formed the basis for subsequent annotation and prioritisation steps (Fig. 2c).

### CNV regions known to be associated with syndromic orofacial clefts

Consensus CNV regions were annotated using AnnotSV and classified according to ACMG guidelines as either pathogenic or variants of uncertain significance (VUS). Variants with Exomiser scores ≥ 0.5 were retained for phenotype-driven prioritisation. Nine probands carried *de novo* CNVs with plausible relevance to OFC pathogenesis (Table 1; Supplementary Table S2). Additional inherited CNVs, though present in unaffected parents, overlapped genes implicated in craniofacial development (Supplementary Table S3). Thus, the integration of phenotypic and molecular data revealed several CNVs directly overlapping known OFC-related genes, including *SHH, WBP11*, *ADAMTS2*, *RXYLY1*, *SC5D*, and *GHR*, all of which have established roles in craniofacial morphogenesis.

### Novel candidate CNV regions for orofacial clefts

Beyond the known OFC-associated loci, several novel CNV regions were detected that may represent candidate genomic contributors to syndromic OFCs (Table 2). These regions encompass genes involved in the 1st branchial arch mandibular and maxillary components, nasal processes, and neural crest development, which are processes integral to craniofacial formation. While their pathogenicity remains to be functionally validated, their recurrent presence in multiple probands and absence in unaffected parents highlights their potential aetiologic significance. Notable candidate CNVs include regions harbouring *RSPH10B2*, *CCZ1B*, *HYDIN*, *ETS1*, *FLI1*, and *PRODH*, all of which warrant further functional investigation to elucidate their roles in craniofacial anomalies.

### Gene expression profiling

**Figure 4:**
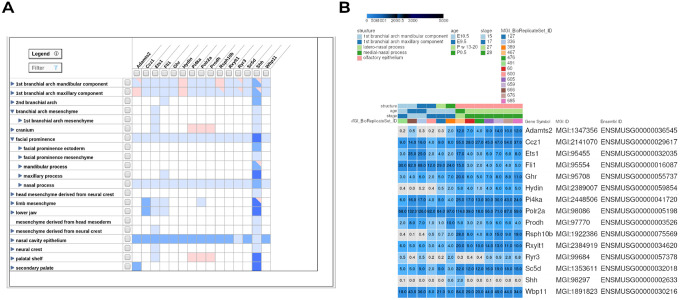
Spatiotemporal expression profiling of high-confidence CNV-associated genes in embryonic craniofacial tissues using Mouse Genome Informatics (MGI). **(a)** Tissue × Gene Matrix illustrating the presence and relative distribution of gene expression for prioritized high confidence genes within CNV regions across embryonic craniofacial structures, including the first branchial arch (mandibular and maxillary components), facial prominence mesenchyme, head mesenchyme, nasal process, maxillary process, palatal shelf, and secondary palate. Expression annotations are shown across relevant developmental stages, highlighting genes expressed in tissues critical for craniofacial morphogenesis. The visualisation uses blue shades to represent expression and red to indicate its absence, whereas blank boxes indicate unavailability of data. The intensity of the colour corresponds to the annotation count: light (1–4), medium (5–50), and dark (more than 50). In cases where structures yielded mixed results, cells are marked with both red and blue triangles. Finally, White cells indicate no annotations for the gene at a particular tissue. **(b)** Heatmap showing transcript-level expression of candidate genes expressed as transcripts per million (TPM) in key craniofacial structures such as the mandibular, maxillary and nasal processes during early embryonic development (E9.5–E10.5). Colour intensity reflects normalized relative expression levels, with higher expression indicated by deeper blue shading. Together, these analyses highlight CNV-associated genes with functional relevance to craniofacial development and syndromic orofacial clefts.

Gene expression profiling using the Mouse Genome Informatics (MGI) Gene Expression Database revealed that several high-priority genes located within the identified CNV regions are expressed in embryonic craniofacial tissues critical for orofacial development (Fig. 4A and 4B). The Tissue × Gene Matrix (Fig. 4A) demonstrated spatially restricted and developmentally regulated expression patterns across key craniofacial structures, including the first branchial arch mandibular and maxillary components, facial prominence mesenchyme, head mesenchyme, maxillary and nasal processes, palatal shelves, and secondary palate.

Notably, *SHH, FLI1*, and *ETS1* showed consistent expression across multiple mouse craniofacial tissues during early to mid-gestation stages, supporting their potential involvement in craniofacial morphogenesis. Several additional genes within the CNV regions also displayed expression in mesenchymal tissues derived from neural crest cells, which are known contributors to facial skeletal and connective tissue formation.

Transcript-level analysis using the heatmap visualisation (Fig. 4B) further corroborated these findings. Expression intensity, reported as transcripts per million (TPM), revealed moderate to high expression of candidate genes in the mandibular, maxillary, and nasal processes between embryonic days E9.5 and E10.5, a critical developmental window for facial patterning and palatogenesis. The coordinated expression patterns observed across these tissues suggest functional convergence of multiple CNV-associated genes during craniofacial development.

**Figure 5:**
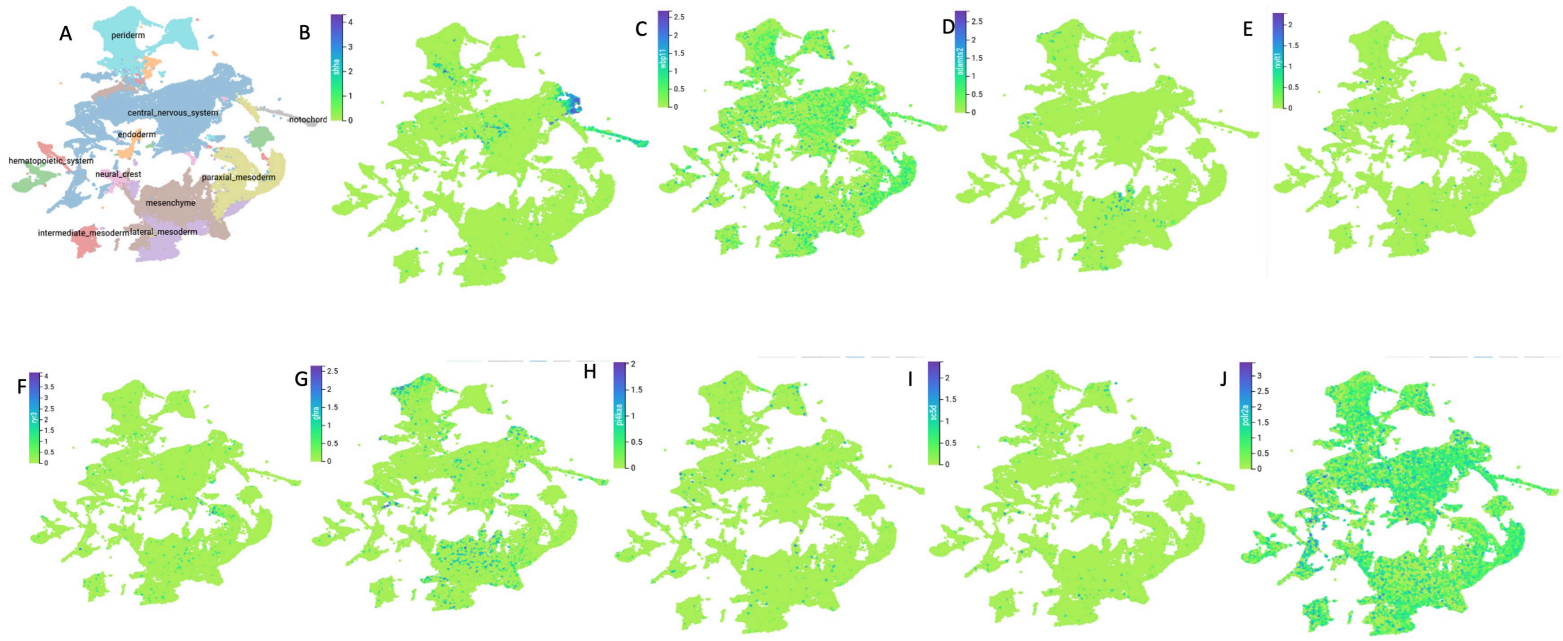
Single cell RNA sequencing spatial gene expression profiles in zebrafish for the implicated genes. (A) UMAP plot showing structural landscape and organization of the zebrafish embryo. (B-J) UMAP plot showing the expression of *Shha, Wbp11, Adamts2, Rxylt1, Ryr3, Ghra, Pi4kaa*, *Sc5d* and *Polr2a* across different cell populations in zebrafish embryo (accessed February 28, 2026). Given the divergent evolution of certain genes in zebrafish, *Shha, Ghra*, and *Pi4kaa* represent the zebrafish orthologs of the human genes *SHH, GHR*, and *PI4KA*, respectively. Expression levels are indicated by color intensity, with darker blue/purple representing higher expression and light green representing lower expression.

**Figure 6:**
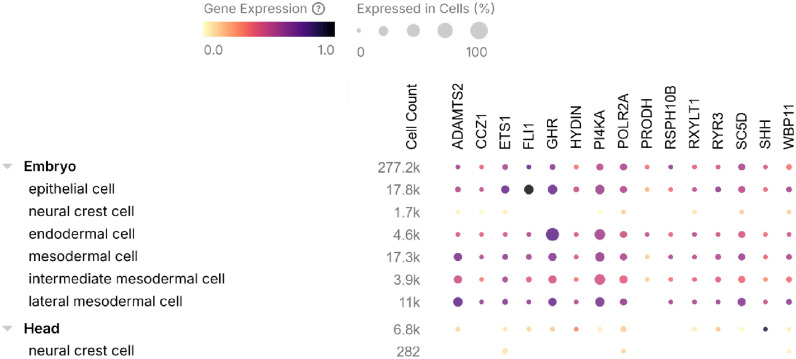
Single cell RNA-seq expression profiles of implicated genes across embryonic and craniofacial structures in human embryos. Expression dot plot illustrating the single-cell RNA-seq expression profiles of candidate genes across human embryonic and craniofacial tissues, derived from the CELLxGENE Discover (accessed February 28, 2026). For each gene-tissue combination, dot size represents the percentage of cells expressing the gene and color intensity reflects the mean expression level among expressing cells, providing a comprehensive overview of the spatiotemporal expression landscape of implicated genes during craniofacial development.

Further, single cell gene expression studies in both zebrafish and human embryos demonstrated that the implicated genes are expressed in cell lineages that are crucial for craniofacial morphogenesis (Figs. 5 and 6; Supplementary Figure S3). For example, *Shha, Wbp11, Adamts2, Rxylt1, Ryr3, Ghra, Pi4kaa, Sc5d* and *Polr2a* were differentially expressed in critical cell lineages such as the periderm, mesenchyme, as well as paraxial, lateral and intermediate mesoderms in zebrafish. In human embryos, these genes are also expressed in various embryonic cell lineages (e.g., epithelial, neural crest, endodermal, mesodermal, intermediate mesodermal and lateral mesodermal cells), as well as cell lineages specific for the head (e.g., neural crest cells).

### Functional Enrichment and Interaction Analysis for detected CNV regions

**Figure 7:**
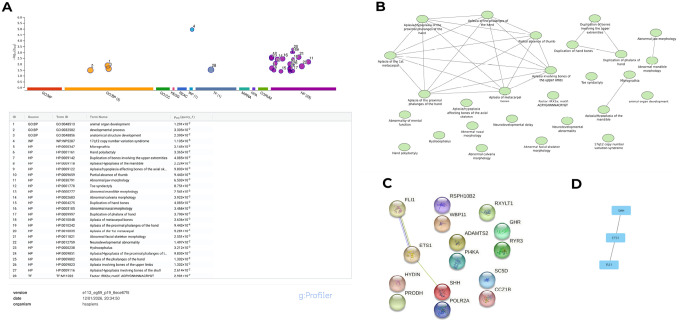
Functional Enrichment and Interaction Analysis **(a)** Pathway enrichment analysis showing significantly enriched terms identified using g:Profiler **(b)** Network visualisation of enriched terms illustrating shared genes and phenotypic overlaps using Cytoscape **(c)**Protein-Protein Interaction network of prioritised genes using STRING Database **(d)**Identified ‘Hub genes” from the PPI network using Cytoscape.

Pathway enrichment analysis was conducted using g:Profiler on genes within *de novo*, candidate, and inherited CNV regions showing Exomiser scores ≥ 0.5. The enrichment was performed under default parameters, with multiple-testing correction using the g:SCS method. Only terms with an adjusted p-value < 0.05 were considered statistically significant. The analysis revealed several key Gene Ontology (GO) biological processes enriched among the implicated genes, notably developmental process (GO:0032502) and anatomical structure development (GO:0048856), underscoring the developmental relevance of the identified CNVs (Fig. 7A). Enriched Human Phenotype Ontology (HPO) terms predominantly reflected craniofacial, neurodevelopmental and skeletal abnormalities, including “abnormal mandible morphology”, “micrognathia”, “abnormal calvaria morphology,” “neurodevelopmental abnormality,” “hydrocephalus,” “toe syndactyly,” and “hand polydactyly.” The gene-sets for each enriched term are summarised in Supplementary Table S4. Figure 7B presents a graph-based representation of the enriched terms, illustrating shared genes and phenotypic overlap among craniofacial, limb, and neurodevelopmental features.

The interactome analysis using the STRING database identified a connected cluster of genes comprising *SHH, ETS1* and *FLI1* (Fig. 7C, D). This cluster represents a core interaction network within the set of CNV-affected genes, implicating coordinated molecular pathways involved in craniofacial development. Perturbations in any of these key genes have the potential to alter regulatory processes essential for normal craniofacial morphogenesis.

## DISCUSSION

This study applied WES data together with the phenotypic characteristics of probands to detect and characterise CNVs associated with syndromic OFCs in a Ghanaian case parent trio cohort. Although WES is primarily used for identifying SNVs and small indels, our findings reaffirm its emerging potential for CNV discovery when analysed using complementary algorithms. By integrating results from multiple CNV callers and phenotype-driven prioritisation, we identified both known and novel, *de novo* and inherited CNV regions potentially implicated in craniofacial development and syndromic OFCs.

Cleft palate (CP) was the most frequent OFC subphenotype in the study cohort, with a slightly higher occurrence in females than males (Table S1). This pattern is consistent with reports that CP exhibits a stronger syndromic component and a female predominance, whereas cleft lip (CL) and cleft lip with palate (CLP) are more common in males [47–49]. These gender-based differences may reflect sex-specific timing in palatal fusion, as delayed palatal shelf elevation in females increases their susceptibility to CP [10]. PRS emerged as the most common recognisable syndrome, consistent with previous reports across various populations [1, 50, 51].

Our analyses buttress the assertion that reliable CNV detection in WES requires integrating multiple algorithms to account for inherent variability across tools. cn.MOPS exhibited the highest overlap rate (69.9%), suggesting greater specificity, whereas ExomeDepth produced more calls with lower concordance, likely due to sensitivity to read-depth noise [35]. Filtering CNVs based on ≥ 50% reciprocal overlap across tools enhanced confidence and minimised false positives, consistent with a study [22]. After stringent filtering, 2,980 high-confidence CNVs were retained, spanning a broad range of genomic sizes except those > 10 Mb, which are typically underrepresented in WES-based detection.

Several CNVs harboured genes with well-established roles in craniofacial morphogenesis (Table 1), supporting their likely pathogenicity in OFC syndromes. A 5.2 Mb deletion harbouring the *SHH* gene was identified in patient GH20140599.1. *SHH* deletions are associated with clinical features such as holoprosencephaly and midline facial defects, reflecting disruption of Hedgehog signalling, a pathway critical for forebrain and facial development [52, 53]. Consistent with this molecular observation, this proband in our cohort was clinically diagnosed with holoprosencephaly, presenting with left unilateral talipes equinovarus, microcephaly, developmental delay (e.g., no neck control at 8 months), heart defect (heart murmur), large, low-set ears, left complete CL and alobar holoprosencephaly (Table S1). Patient GH20207072.1 harboured a 147 kb pathogenic deletion involving the *WBP11* gene. Variants in *WBP11* have been implicated in VACTERL syndrome, a multisystem disorder featuring vertebral, cardiac, tracheoesophageal, renal, and limb malformations, along with craniofacial dysmorphism, growth delay, optic anomalies, and pubertal retardation resembling hypopituitarism [54]. Evidence from animal models, including data from the Mouse Genome Informatics database (https://www.informatics.jax.org/),) underscores the role of *WBP11* in craniofacial development. The proband harbouring this CNV presented with syndromic Pierre Robin Sequence (PRS), with clinical presentations including parietal encephalocele, micrognathia, left microphthalmia, malformed right ear, cleft lip and palate (CLP), and respiratory distress syndrome (Table S1).

Patient GH20228117.1 presented with a 637 bp pathogenic deletion partially overlapping the *ADAMTS2* gene. Emerging evidence suggests that extracellular metalloproteinases, including *ADAMTS* family members, are essential in neural crest cell development. Disruptions in these metalloproteinases can lead to malformations of craniofacial and other neural crest-derived structures [55]. *ADAMTS2* participates in facial bone formation through the ErbB signalling pathway, which is critical for osteoblast differentiation and bone metabolism [56]. Zebrafish models with *ADAMTS2* knockdown exhibit suppressed *Egfr* expression, resulting in craniofacial defects such as an elongated snout, underdeveloped jaw, and CP [56]. The proband presented with right complete CLP and right hexadactyly (Table S1). *PI4KA* duplications observed in patients GH20172509.1 and GH20218075.1 may underlie the CP, mandibular hypoplasia, and syndromic PRS-like features observed in both patients, as well as the global developmental delay in proband GH20172509.1 (Table S1). While *PI4KA* is primarily associated with neurodevelopmental and gastrointestinal phenotypes, emerging clinical data have documented micrognathia and other craniofacial dysmorphisms as part of the broader *PI4KA*-related disorder spectrum [57–59].

Patient GH20218033.1 carried duplications involving the *SC5D* and *POLR2A* genes. The *SC5D* gene, essential for cholesterol biosynthesis, is associated with lathosterolosis, a condition characterised by growth retardation, craniofacial malformations, and CP [60]. *POLR2A* mutations have been linked to neurodevelopmental and craniofacial defects [61]. Interestingly, this proband presented with CP and ankyloglossia (Table S1). A 550bp duplication partially overlapping the *RYR3* gene was observed in patient GH20135043.1. Although *RYR3* is not the predominant isoform of ryanodine receptors, it is often co-expressed with *RYR1* or *RYR2* across various tissues, where it modulates their functional properties [62]. Dysregulation of RyR-mediated calcium signalling is known to disrupt muscle fibre maturation and craniofacial morphogenesis, particularly during early development [63]. Patient GH20160169.1 showed a 564 bp duplication partially overlapping the *GHR* gene. The growth hormone receptor (GHR) plays a pivotal role in mediating the effects of growth hormone, which is integral to craniofacial development. Variations in *GHR* have been associated with differences in mandibular morphology and overall facial skeletal architecture across diverse populations [64]. Characteristic craniofacial features in children with growth hormone deficiency include reduced cranial base dimensions, retrognathism, and a smaller posterior facial height relative to the anterior [65].

Several other *de novo* CNVs were detected in syndromic OFC patients, overlapping genes with limited or no previous association with craniofacial anomalies, yet show developmental relevance based on functional annotations and expression data. Patients GH20130774.1 and GH20130799.1 exhibited 34kb and 28kb *de novo* duplications, respectively; patient GH20134969.1 presented a 19kb *de novo* deletion; GH20207012.1 carried a 44kb *de novo* deletion; and GH20207031.1 harboured an 18kb *de novo* deletion, all located on chromosome 7 and encompassing the genes *RSPH10B2* and *CCZ1B*. Additionally, patient GH20140607.1 showed a 16kb *de novo* deletion, and patient GH20140628.1 showed a 16kb *de novo* duplication overlapping *RSPH10B2*. While neither *RSPH10B2* nor *CCZ1B* has been previously associated with OFCs or craniofacial malformations, developmental expression data from mouse models reveal their expression in embryonic ectoderm, mesoderm, branchial arches, musculoskeletal system, nervous system, and limbs—tissues that are critical to craniofacial morphogenesis. These findings suggest a putative role for *RSPH10B2* (https://www.informatics.jax.org/marker/MGI:1922386, accessed January 13, 2026) and *CCZ1B* (https://www.informatics.jax.org/marker/MGI:2141070, accessed January 13, 2026) in early craniofacial development. Further functional experiments are warranted to validate their contributions to craniofacial morphogenesis and syndromic OFCs.

Patient GH20140628.1 carried a 35kb *de novo* duplication overlapping *HYDIN*, a gene involved in the structure and motility of motile cilia. Pathogenic variants in *HYDIN* are associated with primary ciliary dyskinesia-5 (https://www.genecards.org/cgi-bin/carddisp.pl?gene=HYDIN, accessed January 13, 2026), a disorder involving dysfunctional cilia. Mouse studies demonstrate embryonic *HYDIN* expression in neural tissues, where it participates in processes such as cytoskeletal organisation, cell differentiation, and system development (https://www.alliancegenome.org/gene/HGNC:19368#function---go-annotations, accessed May 27, 2025). Notably, phenotypes associated with *HYDIN* mutations include abnormal cranium morphology and widened cranial sutures (https://www.informatics.jax.org/diseasePortal/popup?isPhenotype=true&markerID=MGI:2389007&header=craniofacial, accessed January 13, 2026), indicating possible involvement in craniofacial patterning. Although not previously linked to OFCs, these data support the candidacy of *HYDIN* for further exploration in craniofacial genetics.

Patient GH20218033.1 harboured a 111kb *de novo* duplication overlapping *ETS1* and a 410kb duplication affecting *FLI1*. *ETS1* is a transcription factor essential for neural crest cell specification and migration, both of which are central to craniofacial morphogenesis. It contributes to the epigenetic control of BMP signalling [66] and plays a role in extracellular matrix remodelling and epithelial-to-mesenchymal transition, EMT [67], both of which are vital during palatogenesis. Mouse expression data show *ETS1* expression in the ectoderm, mesoderm, mesenchyme, pharyngeal arches, nervous system, and musculoskeletal system (https://www.alliancegenome.org/gene/MGI:95455#expression, accessed January 13, 2026). *FLI1*, another *ETS* family transcription factor, shares overlapping functions with *ETS1* and is also hemizygously deleted in Jacobsen syndrome - a condition characterised by facial dysmorphisms and hearing impairment [68]. Studies in mice with combined *Ets1* and *Fli1* mutations show midfacial anomalies, including small middle ear cavities and malformed nasal bone-cartilage interfaces [69]. These findings underscore a cooperative role for *ETS1* and *FLI1* in craniofacial development and support their involvement in OFC-related phenotypes.

Several patients, including GH20134915.1 (4.3kb *de novo* deletion), GH20207012.1, GH20238200.1 (388kb *de novo* deletion), and GH20218061.1 (388kb *de novo* duplication), harboured CNVs overlapping the *PRODH* gene. This gene encodes proline dehydrogenase, a mitochondrial enzyme involved in proline metabolism, and mutations in this gene cause hyperprolinemia type I [70]. Although mouse models do not show specific craniofacial phenotypes for *PRODH* (https://www.informatics.jax.org/marker/MGI:97770, accessed January 13, 2026), the gene resides within the 22q11.2 chromosomal region frequently deleted in DiGeorge syndrome (DGS), a disorder characterised by CP and related craniofacial anomalies [71]. Thus, while *PRODH* may not independently drive craniofacial defects, its presence within a relevant locus for syndromic OFCs suggests it could contribute to OFC phenotypes in a polygenic or modulatory context.

The enrichment analysis revealed critical biological processes and phenotypic abnormalities that may be influenced by CNVs identified in cases of syndromic OFCs. Key significantly enriched Gene Ontology (GO) Biological Process terms included “developmental process” (GO:0032502) and “anatomical structure development” (GO:0048856), indicating that the implicated CNVs may disrupt pathways essential for embryogenesis and tissue morphogenesis. Enriched Human Phenotype Ontology (HPO) terms in this analysis predominantly reflect craniofacial anomalies (e.g., “abnormal calvaria morphology”, “abnormal facial skeleton morphology”, “abnormal mandible morphology”, “micrognathia”) neurodevelopmental anomalies (“17q12 copy number variation syndrome,” “neurodevelopmental abnormality,” “hydrocephalus”) and limb abnormalities (e.g., “hand polydactyly,” “toe syndactyly,” “aplasia involving bones of the upper limbs”). These findings are consistent with the disrupted craniofacial development that characterises syndromic OFCs. This observation aligns with previous research [12], which highlights the association of syndromic OFCs with defects in cardiovascular, neurodevelopmental, and musculoskeletal systems. These terms closely overlap with the phenotypes observed within our current cohort. For example, PRS, characterised by craniofacial abnormalities such as micrognathia and mandibular hypoplasia, corresponds with enriched terms such as “abnormal mandible morphology” (HP:0000277) and “micrognathia” (HP:0000347) [72]. Similarly, mandibular hypoplasia, facial asymmetry, ocular/auricular malformations, and vertebral malformations align with enriched terms such as “aplasia/hypoplasia of the mandible” and “aplasia/hypoplasia involving bones of the axial skeleton” and are consistent with phenotypic characteristics of Goldenhar syndrome [73]. Features of midface hypoplasia and micrognathia, characteristic of Treacher Collins Syndrome, also correspond with these terms [74]. Collectively, the HPO terms suggest significant involvement of craniofacial and skeletal anomalies. Additionally, broader developmental processes, including those involved in bone and skull formation, were implicated, consistent with the phenotypic features observed in some patients. These results underscore the role of CNVs in modulating pathways critical to craniofacial development and the associated syndromic phenotypes in our cohort.

While this study provides valuable insights into the CNV landscape underlying syndromic OFCs in a Ghanaian cohort, several limitations should be acknowledged. First, the reliance on WES data inherently restricts CNV detection to coding regions, potentially missing structural variants within non-coding regions that may also contribute to OFC pathogenesis. Additionally, functional validation of the candidate CNVs identified was beyond the scope of this study, but remains a critical next step to confirm pathogenicity. Thirdly, not all phenotypes might have been obvious in the probands at the time of subject recruitment, especially when they were recruited at a younger age. Unfortunately, once the OFC is repaired, many probands rarely attend follow-up evaluations of their clinical presentations. Lastly, a larger sample size of syndromic OFCs will provide a clearer picture of the contribution of CNVs to the aetiology of these conditions. Despite these limitations, the findings significantly advance our understanding of the genetic basis of syndromic OFCs in African populations. The study demonstrates the feasibility of leveraging WES-based CNV detection tools in resource-limited settings and underscores the value of integrative analytic frameworks combining genomic and phenotypic data.

### Conclusion

Together, our findings support the hypothesis that CNVs detectable through WES contribute to the aetiology of syndromic OFCs by disrupting key developmental genes. The identification of pathogenic CNVs overlapping established craniofacial genes, alongside novel candidate loci revealed through exome-based CNV analysis, underscores the complex genetic architecture underlying these conditions. Importantly, this work represents the first WES-based CNV investigation in Ghanaian case parent trios, highlighting the utility of exome data for structural variant discovery and the importance of including underrepresented populations in genetic studies of congenital craniofacial disorders. We also demonstrate an elaborate workflow that employs a consensus approach among multiple CNV detection tools to call potential pathogenic CNVs. In essence, the current study impacts the diagnosis, pathophysiology, genetic counselling, and other clinical management of syndromic OFCs.

## Supplementary Material

Supplementary Files

This is a list of supplementary files associated with this preprint. Click to download.

- AdditionalFile1.docx
- AdditionalFile2.docx
- AdditionalFile3.docx
- AdditionalFile4.docx
- AdditionalFile5.docx
- AdditionalFile6.docx
- AdditionalFile7.docx

## Figures and Tables

**Table 1:** *De novo* CNVs that harbour genes previously associated with syndromic orofacial clefts.

Patient ID	Chr	Start	End	CNV Size (bp)	CNV Type	ACMG Class	Top Gene	Exomiser Score
GH20140599.1	12	153887435	159145021	5257586	DEL	5	*SHH*	0.7396
GH20207072.1	12	14695982	14843363	147381	DEL	5	*WBP11*	0.7369
GH20228117.1	5	179344941	179345578	637	DEL	5	*ADAMTS2* (HI = 30)	0.5145
GH20130815.1	12	63779711	63809342	29631	DUP	3	*RXYLT1*	0.6560
GH20135043.1	15	33863889	33864439	550	DUP	3	*RYR3*	0.5056
GH20160169.1	5	42628789	42629353	564	DUP	3	*GHR*	0.5698
GH20172509.1	22	20709046	20713039	3993	DUP	3	*PI4KA*	0.6915
GH20218033.1	11	120802444	121629813	827369	DUP	3	*SC5D*	0.7610
GH20218033.1	17	7512973	7514429	1456	DUP	3	*POLR2A*	0.6089
GH20218075.1	22	20711100	20713039	1939	DUP	3	*PI4KA*	0.7570

Nine individuals were identified as harbouring *de novo* copy number variants, with Exomiser scores either consistent with the patient’s phenotype (score > 0.5) or highly specific and consistent with the phenotype (score > 0.7). A haploinsufficiency (HI) score of 30 indicates the gene is associated with an autosomal recessive phenotype. Other genes in each of the CNV regions are listed in Supplementary Table S3. *De Novo* CNVs were identified by intersecting the CNVs identified in both the proband and parents and filtering for CNVs which were found only in probands but not in parents. An ACMG score of 5 denotes a pathogenic variant, whereas a score of 3 denotes a variant of uncertain significance (VUS)

**Table 2:** Other *de novo* CNVs in syndromic OFC Cases with potential craniofacial relevance.

Patient ID	Chr	Start	End	CNV Size (bp)	CNV Type	ACMG Class	Gene(s)	Other Genes in the Region
GH20130774.1	7	6,780,559	6,815,095	34,536	DUP	3	*RSPH10B2, CCZ1B*	None
GH20130799.1	7	6,796,319	6,824,594	28,275	DUP	3	*RSPH10B2, CCZ1B*	None
GH20134969.1	7	6,780,559	6,799,529	18,970	DEL	3	*RSPH10B2, CCZ1B*	None
GH20207012.1	7	6,780,560	6,824,594	44,034	DEL	3	*RSPH10B2, CCZ1B*	None
GH20207031.1	7	6,780,559	6,799,529	18,970	DEL	3	*RSPH10B2, CCZ1B*	None
GH20140607.1	7	6,780,559	6,797,016	16,457	DEL	3	*RSPH10B2*	None
GH20140628.1	7	6,780,559	6,797,016	16,457	DUP	3	*RSPH10B2*	None
GH20140628.1	16	70,943,562	70,979,291	35,729	DUP	3	*HYDIN*	None
GH20218033.1	11	128,462,112	128,573,380	111,268	DUP	3	*ETS1*	*MIR6090, ETS1-AS1, LOC124902790, LOC105369565*
GH20218033.1	11	128,781,709	129,192,448	410,739	DUP	3	*FLI1*	*KCNJ1, LOC107984409, KCNJ5, KCNJ5-AS1, TP53AIP1, ARHGAP32*
GH20134915.1	22	18,912,925	18,917,238	4,313	DEL	4	*PRODH*	None
GH20207012.1	22	18,528,750	18,917,238	388,488	DEL	4	*PRODH*	*TMEM191B, PI4KAP1, LOC124905077, LOC124905174, LOC124900482, RIMBP3, FAM246B, FAM230E, FAM247C, GGT3P, POM121L15P, LOC102724728, FAM230F, DGCR6*
GH20218061.1	22	18,528,750	18,917,238	388,488	DUP	3	*PRODH*	*TMEM191B, PI4KAP1, LOC124905077, LOC124905174, LOC124900482, RIMBP3, FAM246B, FAM230E, FAM247C, GGT3P, POM121L15P, LOC102724728, FAM230F, DGCR6*
GH20238200.1	22	18,528,750	18,917,238	388,488	DEL	5	*PRODH*	*TMEM191B, PI4KAP1, LOC124905077, LOC124905174, LOC124900482, RIMBP3, FAM246B, FAM230E, FAM247C, GGT3P, POM121L15P, LOC102724728, FAM230F, DGCR6*

All CNV regions were *de novo*. All genes had Exomiser scores below the threshold of 0.5 except for *ETS1* and *FLI1* with scores of 0.5697 & 0.5119, respectively. An ACMG score of 5 denotes a pathogenic variant, 4 denotes likely pathogenic, whereas a score of 3 denotes a variant of uncertain significance (VUS)

## Data Availability

The whole exome sequencing dataset reported in this article can be accessed through the FaceBase Consortium: Gowans LJJ. Clinical Deep Whole Exome Sequencing of a Syndromic Orofacial Clefts Cohort from Ghana (DECIDE). FaceBase Consortium 2025, with accession number 94-D420. The informed consent obtained from participants only permits sharing the WES dataset under controlled access. The data and materials that support the findings of this study are available from the corresponding author upon reasonable request.

## Abbreviations

BAM: Binary Alignment/Map
CL: Cleft Lip
CLP: Cleft Lip and Palate
CNV: Copy Number Variant
CP: Cleft Palate
EEC: Ectrodactyly, Ectodermal Dysplasia, Clefting
GO: Gene Ontology
HP: Human Phenotype
HPO: Human Phenotype Ontology
Indels: Insertions/Deletions
OFC: Orofacial Cleft
PRS: Pierre Robin sequence
SNV: Single Nucleotide Variant
SAM: Sequence Alignment/Map
VWS: Van der Woude syndrome
WES: Whole Exome Sequence
WGS: Whole Genome Sequencing
